# The Construction of Artificial Crowns

**Published:** 1901-05

**Authors:** James P. Nichol

**Affiliations:** Philadelphia


### THE CONSTRUCTION OF ARTIFICIAL CROWNS.[4]

BY JAMES P. NICHOL, D.D.S., PHILADELPHIA.

Mr. President and Gentlemen,—It is not my intention to attack any established rules or theories this evening in regard to the methods you have already employed.

I have been asked to present a paper to you, taking for my subject "The Construction of Artificial Crowns," and calling attention to some practical points in their manufacture.

I esteem it an honor to come before this society, and being an active student in this line of work, I feel that in any discussion that

---

[4] Read before the Academy of Stomatology, Philadelphia, February 26, 1901.

might follow we may be able to get some ideas that will be a benefit to all of us.

Let us take the Richmond crown, for instance. You are all familiar with the construction of this style of crown. Allow me to ask, Why should such heavy backings be used? We all know there is absolutely no union between platinum, or other metal used for backing, and the porcelain facings.

Now, if soft platinum about 1/1000 gauge (same as is used in inlay work) is used and burnished nicely around all the edges after soldering and finishing the piece, we get splendid margins, and the danger of checking facings, so far as tight backings are concerned, is almost eliminated.

The method I have been using in backing a Richmond incisor is as follows: cut the backing a trifle larger than the facing all around; on the approximal surfaces let it overlap about a line, on the cutting edge leave it, say, about one-sixteenth to one-eighth of an inch long; by snipping it along this edge at intervals the wrinkling may be avoided, as it conforms to the curves of the facing.

It is well to punch the pin-holes in the backing as near the exact size of the pins in the facing as possible; if they are enlarged, as they usually are, the flux or borax will be drawn through between the backing and facing and will cause checking of the facing. After the backing is burnished nicely the piece is placed on the model and waxed up; this being done, brace the piece with the fingers on the labial or buccal surface, and by pressing on the wax you can crowd the soft thin backing to the facing; thus getting an excellent adjustment. In investing the piece let the material cover the overlapping edges so that when the wax is removed the backing will be held in position by the investing material until the piece is soldered.

In the construction of the Richmond bicuspid crown there is another point to which I would call attention, that is the joint between the cusp and the facing.

To make an artistic piece of work and show the least possible amount of gold, a mitred joint should be used so that the cuspal end of the porcelain is ground at an angle of about forty-five degrees with the back of the facing.

We must not overlook the contraction that takes place, more or less, after soldering a crown of this type, and with this in view we should fit the joint between the cusp and facing accordingly.

After the facing has been bevelled and waxed in position, the cusp is then filed to the opposite bevel of porcelain. In placing it in position it should fit the facing closely at the buccal aspect and stand out slightly at the flat surface, or, in other words, at the backing; in cooling after soldering the contraction would take place almost on a line with the points designated, and the joint be closed.

Another question has been asked many times, "How do we account for the checking of facing?" Let us look over the work in a few stages of its construction and see if we can locate the cause.

It is my opinion that the dentist is at fault in nine cases out of ten. I have tried a simple experiment by taking a facing made by each of the largest tooth manufacturers and placing them in a furnace together with some solder; after heating the furnace to the fusing point of the solder, I shut off the current, removed the facings, and failed to find any checking. I therefore conclude that the checking is not due to the inability of the facing to withstand the high temperature requisite for soldering.

In the first place, the porcelain should not be heated up too much by grinding, as the uneven heating by friction will weaken it. Secondly, the wax should all be removed before the backings are fitted (wash with chloroform). Thirdly, the backings I have already mentioned in detail. Fourthly, the heating up and soldering, and here to my mind is the stage where the greatest amount of checking is done.

We are, of course, well aware that platinum and vitreous materials, like glass and porcelain, have approximately the same coefficient of expansion when subjected to uniform changes of temperature. Practical recognition of this similarity of expansion and contraction is shown in the use of platinum wires as electrical conductors when fused into the bulbs of incandescent electric lamps, as well as its various other forms of vacuum electrical appliances or apparatus; for example, Geissler and Crookes tubes.

It is not the high fusing-point nor the unalterability of platinum which renders it especially useful for this purpose, but the much more important property of its coefficient expansion with that of glass.

It is this property also which, quite apart from its high fusing-point, makes it the most suitable metal to construct pins for porcelain artificial teeth, around which the porcelain body must be fused in order to retain the pins in place.

But while the coefficient of expansion of the platinum and porcelain respectively is practically the same, it must not be forgotten that the relative rates of expansion of these two bodies is directly proportional to their rates of thermal conductivity.

Hence, as the rate of thermal conductivity of platinum is much higher than that of porcelain, it becomes necessary, in order to avoid checking of a porcelain facing, not to heat the facing suddenly, whereby the limit of expansion of the metallic pins is reached before the increase in temperature of the surrounding porcelain material has developed a compensating expansion to offset the strain which would otherwise be produced by the premature expansion of the metallic pins.

It is the too sudden application of heat which, in my judgment, is the most fruitful source of fractured facings in the soldering operation.

After a piece is invested it is not essential to have the case thoroughly dried before heating up; it may be placed on the furnace and warmed up slowly, removing the wax in due time. After the investment turns brown (showing the wax to be burned out), the flame of the blow-pipe is then applied around the outer surface of the investment, but never to the metallic structure,—namely, the pins and backing.

Now, the parts being heated to a point where the solder will flow, if the flux be used judiciously the percentage of checked facings will be reduced to a minimum.

The full gold crown is causing considerable comment at the present time, and several "swaging appliances for seamless crowns" have been placed on the market recently. I will state that this type of crown may be produced absolutely seamless in about half the time it takes to go through the swaging method; there is no danger of splitting the metal in stretching, and a perfect occlusion may also be obtained.

The method, in short, is as follows: Measure the tooth, cut and sweat a band (by sweating I mean the union of the cut ends by the autogenous method of direct fusion). The contouring may be nicely done with heavy short-beaked pliers, stretching or bulging the band out to within one-sixteenth of an inch of the cervix, at which point it passes beneath the free margin of the gum.

After shaping the occlusal margin of the band and making proper indentations for the fissures, a cusp is selected or an impression of the occluding tooth used, and by means of a special stamping device a matrix of pure gold about 2/1000-inch gauge is struck up, and filled with the same karat gold that the band is made from; the cusp is filled true, making contact with the band at every point, and held in position with binding wire; the joint is fluxed, and with the piece lying on charcoal the band and cusp are united without solder by means of the blow-pipe. The result is a seamless crown in which all danger of discoloration is avoided by the absence of the solder; on the other hand, the sweat-joint in the band is more pliable and is equally as strong as any part of the band.

Even though we grant that the result may be practically the same with either method, the swaging process is defective inasmuch as it fails to develop and maintain that high degree of manipulative skill which it should be the ambition of every operator to attain and conserve; it is the difference between the product of the skilled and artistic workman and the automatic machine.

The porcelain art in dentistry, or the method of baking artificial crowns, etc., is one that is also exciting a great deal of interest among our profession. For single crowns on the ten anterior teeth it would seem that here would be its widest range of usefulness, as it is a question whether it will ever replace the all-gold crown or modifications of the same for strength and durability on the molar teeth, certainly not in the very short bite cases, as it is in proportion to the amount of porcelain used in this work that we get the best results in resisting the force of mastication.

I do not mean by this that the metal construction work is to be overlooked in any way, but, porcelain being a fragile substance at best, it depends almost entirely upon the bulk of porcelain body for strength.

I believe that a well-constructed porcelain incisor crown is as strong, and will last as long, as a Richmond crown, in which we are dependent entirely on the pins for strength to hold the facing, but in the baked porcelain crown the body is fused to the surface of the facing and will not break away.

The shade is more easily retained, and this is very often changed considerably by the backing; the facing seldom or never loses any of its translucency, which it does to a certain extent after a backing has been used.

